# WHERE DOES RESPONSIBILITY LIE? ANALYSING LEGAL AND REGULATORY RESPONSES TO FLAWED CLINICAL DECISION SUPPORT SYSTEMS WHEN PATIENTS SUFFER HARM

**DOI:** 10.1093/medlaw/fwac022

**Published:** 2022-07-20

**Authors:** Megan Prictor

**Affiliations:** Melbourne Law School and Centre for Digital Transformation of Health, The University of Melbourne, Carlton, Australia

**Keywords:** Clinical decision support systems, Consumer law, Contract, Harm, Negligence, Regulation

## Abstract

Clinical decision support systems (CDSSs) are digital healthcare information systems that apply algorithms to patient data to generate tailored recommendations. They are designed to support, but neither dictate nor execute, clinical decisions. CDSSs can introduce new risks, both by design features that heighten clinician burden and by outright errors that generate faulty recommendations for care. In the latter instance, if such unintercepted recommendations were to result in harm to the patient, novel legal questions emerge. Does legal responsibility for this harm lie with the clinician, the software developer or both? What is the clearest path to a remedy? Further, how does the Australian regulatory framework provide for oversight and redress? This article analyses the potential forms of legal redress in negligence, contract and under statutory consumer law, for the patient and the clinician. It also examines the Australian regulatory framework, specifically in relation to the Australian Competition and Consumer Commission and the Therapeutic Goods Administration, and reflects on the framework’s adequacy to protect patients and clinicians. It finds that the regulatory approach and the contour of legal risk still centre upon the clinician’s duty to exercise decisional autonomy and to intercept flawed recommendations generated by algorithmic errors within CDSSs.

## I. INTRODUCTION

Clinical decision support systems (CDSSs) are digital information systems in health care settings that ‘enable the comparison of individual patient data with an existing knowledge base, so that algorithms can be used to generate clinical recommendations specific to the patient’.^1^ These tailored recommendations can relate to diagnosis, treatment, the provision of patient information, monitoring, recall, and follow-up. An important feature of CDSSs is that they are designed to *support*, but not to *dictate* nor independently *execute*, clinical decisions. The law requires clinicians to exercise their own autonomous judgement regarding the provision of care to a patient irrespective of the presence of a CDSS.^2^ It is an unassailable legal principle that the medical practitioner’s duty encompasses the ‘exercise [of] skill and judgment’.^3^ CDSSs are designed to require and support such exercise of professional judgment when applying the system’s recommendations to an individual patient.^4^

When CDSSs are introduced into clinical practice they introduce a new element to the clinical decision-making process. Patients are not users of a CDSS; they ought to encounter its effects mediated entirely by the clinician. Nonetheless, their care may be influenced by the design of a CDSS, potentially to a great extent. If these systems work correctly to enhance guideline-concordant care and reduce errors, this new element raises no new legal issues. As previously shown,^5^ the clinician will be supported in acquitting their existing duty to the patient through the readier availability of relevant information. The likelihood of the patient receiving harmful health care should be reduced. However, if the CDSS is flawed in a particular way that means it delivers erroneous recommendations to the clinician user which, unintercepted by them, results in harm to a patient, novel legal questions emerge. Does legal responsibility for this harm lie with the clinician, with the software developer,^6^ or both? What is the clearest path to achieve redress for the patient? There may also be harms to the clinician’s reputation and employment, as well as their mental state. How, in law, can these be mitigated? Further, does the Australian regulatory framework currently offer avenues for oversight and redress in this environment? These questions—the subject of this article—have not been considered in detail to date in the Australian context, although Smith and Fotheringham recently evaluated similar issues with reference to the UK.^7^ The significant growth of the CDSS industry and adoption of these products in Australian health care settings makes it timely to address these questions in this article.

The article will commence with an outline of the types of harm that might be caused by a flawed CDSS. It will present a doctrinal analysis of the potential legal redress available to patients, and then to clinicians, seeking to navigate the challenges inherent in the ‘entwined’ nature of clinical practice using CDSSs.^8^ The latter part of the article will consider the current options available to regulators of CDSSs and whether they are sufficient to protect against the risk of harm and maximise beneficial outcomes in the event such harm should occur.

## II. WHAT HARMS MIGHT CDSSs CAUSE?

The literature on CDSSs highlights their generally positive effects on professional behaviours and some patient outcomes. These effects include reducing medication errors,^9^ improving timely diagnosis,^10^ and enhancing clinician adherence to appropriate processes of care, such as ordering or prescribing recommended treatment,^11^ undertaking preventive care services,^12^ providing vaccinations, and ordering tests.^13^ Nonetheless, serious clinical lapses have also been attributed to the use of these systems, some of which have resulted in harm to patients. Wright and others found, in their 2016 analysis, indications that ‘CDSS malfunctions are widespread, that they likely occur much more frequently than has previously been described, and that existing detection systems (including testing processes, monitoring procedures, and feedback systems) are inadequate to detect CDSS malfunctions before they reach users’.^14^ Broadly speaking, such malfunctions fall into two different categories: clinical errors that are induced or promoted by the design of the CDSS user interface (but are not attributable to system flaws), and those driven by errors in the CDSS itself. I will briefly outline the former before turning to the latter; the main focus of this article.

### A. *Errors Induced or Promoted by System Design*

Errors in the provision of patient care might be attributable directly to a clinician’s act that is induced by poor interface presentation within a CDSS tool. In this class of errors, there is no obvious mistake in the software, nor any wrong advice being generated. Rather, unintended consequences may flow from the way in which the clinician interacts with the system interface. Ash and colleagues identified this class of errors almost two decades ago and have described a range of causes.^15^ For example, alerts for drug–drug interactions generally indicate that a CDSS is functioning correctly; that is, the alerts are the intended output of a well-built system. However, alert fatigue is a known consequence of such systems. This can lead to clinicians disabling or ignoring alerts in between 49% and 96% of cases^16^; a practice which can, in turn, mean that potentially dangerous drug–drug interactions are overlooked, leading to patient harm.^17^ Other types of unintended consequences of CDSS systems, when they are working as they ‘should’, include erroneous entries through auto-complete or simply through the clinician taking insufficient care,^18^ and system rigidity, such as fixed options within lists or difficulty in viewing previous pathology requests.^19^ Improvements in interface design might reduce or eliminate such effects. Even though elements of CDSS such as pop-up alerts might increase the generalised sense of mental burden for clinicians, it would be hard to attribute legal liability for clinician error to software developers in such instances, not least because causation would be difficult to prove.

### B. *Errors within the CDSS*

The focus of this article is the balance of legal duties attributable to software developers and clinicians, and the role of regulators, when a CDSS inadvertently makes incorrect recommendations—for instance through an error in the source information or a failure to update CDSS recommendations in line with changes in source information—or when a CDSS otherwise fails to work as intended. If these types of errors reach a threshold of clinical significance, the clinician must identify and deliberately counteract them to avoid causing harm to a patient. Ash and colleagues described these as problems related to CDSS’ algorithmic content—content that was wrong, misleading or not up-to-date.^20^ An example might be when a clinical guideline has been updated by a peak body, or a new contraindication for a common medicine is promulgated by the drug manufacturer, but these updates are not adopted promptly within the CDSS.^21^ In one real-world instance, a CDSS designed to provide ‘supporting, warning, and blocking interventions’ for high-alert medications (‘HAMs’: medications more likely than others to cause significant patient harm) was introduced to Korea’s largest acute care hospital.^22^ Although the CDSS did reduce occurrences of medication orders exceeding the maximum-dose cut off, there were still nine instances of this dangerous event during the study period post CDSS introduction. The authors noted ‘[r]e-examination of the program revealed an error. After it was corrected, no further maximum-dose-exceeding HAM orders were found again’,^23^ although instances were identified of other warnings failing to fire in certain circumstances. A clinician relying on this system to prevent medication errors could have received false reassurance about inappropriate, perhaps harmful, prescribing choices.

Errors in CDSSs may develop and accrue over time, for instance as external clinical guidelines and medication alerts are updated and these changes must be incorporated within the software. Some changes to external parameters may be subtle and a failure to adopt them promptly within a CDSS may introduce little risk of significant harm to patients. Other updates may be urgent, however, such as product recalls and hazard alerts issued by Australia’s Therapeutic Goods Administration (TGA). To ensure that CDSSs are trustworthy, software developers should have robust systems for determining when updates are needed, for integrating these and testing their operation, for encouraging users to install available updates, and for enabling users to identify the source and currency of a CDSS’s content if they wish. Maintaining the algorithmic currency of CDSSs is a complex and demanding undertaking; as Stone notes, ‘[i]deally, the CDSS team would keep abreast of all of these changes to external systems and do regression testing prior to upgrades … Considering the number of external systems, the number of changes to these systems, and the siloed organizational structure of most complex health care organizations, this is a daunting task’.^24^ Research confirms the frequency of CDSS errors in health care, with 93% of Chief Medical Information Officers in a recent survey reporting at least one malfunction in these systems.^25^ Alerts failing to fire and firing wrongly are both widespread, leading to undertreatment or overtreatment^26^ and the risk of consequential physical harm to patients. To further explore these issues from a legal perspective, I present a hypothetical scenario followed by a detailed analysis of the potential legal claims. This scenario focuses on the class of errors inherent in the software rather than those induced by an unfriendly user interface.

## III. SCENARIO

This hypothetical scenario draws upon a case report from the United States by Stone, describing an unintended adverse consequence relating to a commercial CDSS deployed as part of the implementation of a large-scale electronic health record system.^27^ Stone’s report has been varied for the purposes of this legal analysis. Important caveats are that the scenario does not outline the clinician’s employment context in any detail, as an analysis of the potential legal liability of the employing hospital or health care service is beyond the scope of this article; nonetheless, in practice, this would be an important consideration. Nor does it involve a distributor as intermediary between the software developer and clinician, although in practice that may commonly occur.

A software company produces a CDSS that is intended for wide use in Australian health care settings. It is principally designed to make recommendations for appropriate prescribing, in line with clinical guidelines from national or peak bodies (such as the Heart Foundation or Kidney Health Australia). The CDSS also relies upon a third-party pharmaceutical database as an external knowledge source that is interpreted by software engineers and embedded into algorithms that form part of the CDSS. In this instance, the third-party database classifies certain hypertension medications into three groups: (A) alpha-blockers, (B) beta-blockers, and (C) medicines with both alpha- and beta-blocking effects. The items in category (C) are not listed in categories (A) or (B).

A software engineer writes logic for a medication alert in the CDSS with the assumption that medicines in category (C) (with dual effect) are also listed in categories (A) and (B), which would prevent a situation whereby a patient is prescribed both an alpha/beta blocker and an additional (unnecessary) alpha *or* beta blocker. This assumption was incorrect. For the purposes of this scenario, the operation of this alert is not tested by anyone else or subject to any additional checks before the software is deployed to clinical settings. The software developer provides clinicians with training in how to use the software. It does not provide any accompanying information about the provenance of the algorithms which generate the CDSS recommendations,^28^ the testing and quality assurance processes the CDSS has undergone, the date of last update, nor any other information which might indicate to interested clinicians the extent to which they can safely rely upon the software.

When a patient is admitted to the health care service suffering a heart attack, they are prescribed a medication in category (C), that is, it has both alpha- and beta-blocking effects. The patient subsequently recovers enough to be discharged from hospital. Immediately before they are discharged, a clinician acts on a CDSS alert for the patient, based on a guideline that recommends commencement of a beta blocker in patients who had a heart attack and who had not already been prescribed a beta blocker. Since the CDSS system logic does not consider the existing medication to be a beta blocker (but rather considers it an ‘alpha and beta blocker’ from category (C)), the clinician prescribes a(nother) beta blocker (category (B)). The patient is then effectively prescribed two concurrent beta blockers and dies at home 3 days later of a beta blocker overdose. The clinician experiences severe psychological distress and is fearful about what this outcome means for their career, including potential disciplinary action and loss of employment.

This scenario gives rise to a wide range of options for legal redress that may be available to the parties—both the clinician and the patient’s estate. I will first analyse the potential claims by the patient’s estate against both the clinician and the software developer, seeking to answer the question posed earlier about where responsibility for patient harm is likely to fall. I will also consider whether the clinician can claim against the software developer. This analysis will range across the law of negligence, contract, consumer, and product liability law. It will go on to consider the role of key regulators, the Australian Competition and Consumer Commission (ACCC) and the TGA.

## IV. CLAIMS BY THE PATIENT’S ESTATE

### A. *Potential Claims against the Clinician: What Role Does the Software Error Play?*

#### *1.* Negligence

For the purposes of this hypothetical scenario, the patient has died because of the clinician’s mis-prescribing. The patient’s estate^29^ sues the clinician for negligence. The clinician owed a duty of care to the patient^30^ to prescribe medication appropriately. It appears that the clinician breached this duty of care in prescribing two drugs both having a beta-blocking effect, and that this breach caused the patient’s death.

There is, however, at least a degree of legal uncertainty in relation to this posited breach of duty, since the clinician was relying upon the advice of a CDSS whose design fault caused (or at least contributed to) the harm. As Smith and Fotheringham state, ‘it is difficult to discern the relative influence of the human clinician and the non-human AIS [artificial intelligence system] … the clinician may act as an independent, knowledgeable intermediary … but in practice is encumbered with the responsibility for computer-generated clinical advice over which they have only limited influence’.^31^ Much will turn on the court’s assessment of whether the risk of harm was foreseeable^32^; that is, did the clinician know, or ought they have known, that they should have taken the precaution of cross-checking the patient’s medication record or a similar protective step, instead of relying on the CDSS alone? In making this assessment, the court would consider the probability that the harm would occur if care (such as cross-checking the medication record) were not taken^33^; which takes into account the likelihood that a CDSS will contain a fault of this nature. The court would also weigh up the burden of taking precautions to avoid the risk of harm^34^; that is, how difficult it is to cross-check a medication record, and the burden of other potential precautions such as review by a pharmacist.

A clinician facing this legal claim may raise the defence of peer professional opinion,^35^ whereby they would seek to adduce expert evidence that reliance on a CDSS is widely accepted in Australia by a significant number of respected practitioners in the field as competent professional practice in the circumstances. However, it is improbable that respected practitioners would testify that absolute reliance upon a CDSS tool represents competent practice. There would likely be a focus on expert evidence as to the error rate of CDSSs and the accepted practice of respected clinicians in their degree of reliance upon these systems. While the particular facts of an individual case will be key to any judicial decision, the type of event described above would probably be accepted by a court as a breach of the clinician’s duty of care to the patient.^36^ The crucial point is that CDSSs are designed to provide decision *support*, rather than to directly act upon a patient without expert intervention. While CDSSs are increasingly nuanced and targeted, both the known error rates described earlier and the intended operation of these systems’ recommendations via an intermediary means it is unlikely that unhesitating reliance on the software would be considered acceptable practice. The logical, if alarming, consequence of this analysis is that the use of a CDSS introduces a new source of legal risk for clinicians; they must be on guard against the very system that is put in place to assist them.

#### *2.* Breach of Contract between Patient and Clinician

As an alternative to and concomitant with an action in negligence, a patient might also take action against a clinician for breach of contract. No express contract needs to exist. As Brennan CJ noted in *Breen v Williams*, ‘[i]n the absence of special contract between a doctor and a patient, the doctor undertakes by the contract between them to advise and treat the patient with reasonable skill and care’.^37^ This duty, which is concurrent with the duty in negligence, operates as an implied term of the contract. This line of analysis follows that of tort so closely that it need not be considered further here^38^; it is likely that liability for the breach would fall upon the clinician.

#### *3.* Breach of Australian Consumer Law: ‘Due Care and Skill’

A further cause of action for patients could be the breach of statutory guarantees under the Australian Consumer Law (ACL),^39^ that services will be ‘rendered with due care and skill’ (section 60). This does not require the existence of a formal contract. In the given scenario, the clinician’s action in wrongly prescribing the medication would be treated as a potential breach of the ‘due care’ element. This language draws upon the statutory regime that preceded the ACL, the *Trade Practices Act 1974* (Cth), and requires that services be rendered in a ‘careful, skilful and workmanlike way’.^40^ The breach of duty clauses in the state-based torts legislation (eg *Wrongs Act 1958* (Vic) section 48) are relevant in considering whether there has been a breach of the consumer guarantee.^41^ Hence, a breach of duty in negligence will likely also be a breach of the ACL provision. This would be considered a ‘major failure’ under the legislation (section 268, for instance, where the ‘supply of services creates an unsafe situation’ at paragraph (e)), and the patient’s estate would be able to claim compensation.^42^ Madden has noted that the ACL consumer guarantees cannot be contractually waived—any such waivers being void under section 64.^43^

So far, this analysis of the hypothetical scenario resembles any typical medico-legal claim where a clinician’s error has led to patient harm. The fact of the clinician’s reliance upon the CDSS (at least to some extent) introduces new possibilities for legal redress against the software developer both by the patient and clinician. The following analysis will consider the extent to which the software developer owes a duty of care to the patient (and at Section V, to the clinician) and whether the ACL might provide a better avenue for their claims.

### B.* Potential Claims against the Software Developer by the Patient’s Estate*

The patient’s estate could pursue the software developer directly (alternatively or in addition to their claim against the clinician). Alternatively, the defendant clinician may join the software developer as a second defendant, arguing joint liability for the harm the patient suffered. There are greater impediments to a successful negligence action by the patient’s estate against the software developer than against the clinician, since the patient will need to establish, on the balance of probabilities, the existence of a duty of care between the parties—a duty that is already well established as between clinician and patient. Below are considered a negligence claim against the software developer, followed by an analysis of a claim by the patient’s estate under section 138 of the ACL.

#### *1.* Negligence

##### a. Duty of Care

For this element of the negligence claim to be made out, the patient’s estate must demonstrate that the software developer ought reasonably to have foreseen that their conduct may be likely to cause the plaintiff (or a class of persons to whom the plaintiff belongs, ie other patients) loss or damage. There is no established duty of care between a medical software developer and patient. The plaintiff might derive support from the analogous duty of a manufacturer to their ultimate consumer first recognised in *Donoghue v Stevenson*^44^ and adopted in Australia in *Australian Knitting Mills v Grant.*^45^ However, the facts of this matter would almost certainly be distinguished; here the intended end user (‘ultimate consumer’) of the software is the clinician, not the patient. It is likely, then, that the plaintiff would need to establish a novel duty,^46^ by arguing that the ‘salient features’ of the facts in the case mean that it is appropriate to impute a legal duty upon the software developer to take reasonable care.^47^

The extensive list of ‘salient features’ promulgated by Allsop J in the authoritative case of *Caltex Refineries*^48^ guides consideration of this issue, although the degree to which it establishes a practical structure or test for determining the existence of a novel duty of care is questionable.^49^ Weighing in *favour* of the existence of a duty of care from the software developer to the patient would be: that the nature of the harm alleged is serious (that is, the patient died); and it is reasonably foreseeable (not ‘farfetched or fanciful’^50^) that a fault in a CDSS could cause harm to a patient, since CDSS recommendations are designed to ‘reach’ the patient, albeit through the action of the clinician. Smith and Fotheringham go further here, suggesting that the fact that the patient’s data are processed within the CDSS establishes the necessary relationship and gives rise to a duty of care^51^ since the harm would be reasonably foreseeable. Further, the software developer could take steps to avoid this harm, for instance by implementing a well-developed quality assurance and testing process. Arguably, the nature of the activity undertaken by medical software developers (ie designing software that impacts patient care), means that they should exercise care with a view to avoiding harm to patients.

Several features of the scenario counter this, and weigh against the establishment of a novel duty of care. Defence arguments would include that the patient relies not upon the CDSS but upon the clinician interpreting it, and the software developer is unable to exercise control over the clinician’s intervening conduct. Further, it would be posited that there is neither proximity nor an existing relationship between the software developer and the patient, although the CDSS’s use of patient data, mentioned above, could threaten this argument. This issue of proximity is likely the key in this analysis overall. It is an open question whether a reasonable person in the position of the software developer would foresee that their carelessness would be likely to cause harm to the patient,^52^ when CDSS are always designed to involve a clinician interpreting the system recommendations and selecting only those appropriate to the specific patient and clinical context. Typical CDSS design features, whereby alerts can be dismissed by clinicians, adds weight to this view; if the clinician were instead forced by the system to follow its guidance without the opportunity for workarounds or independent action, it would be easier to establish that the software developer was sufficiently proximate to the patient to owe them a duty of care.

There is also an important policy consideration here. If software developers do owe a duty of care to patients, are clinicians consequently absolved of responsibility in this specific area of clinical decision-making? It seems inappropriate to carve out an element of clinical practice where the clinician does not owe their patient a duty to exercise reasonable care and skill, in a scenario of this type. Only if the machine became solely ‘responsible’ for clinical decisions and can give them effect directly, would it be fitting to give preference to the manufacturer’s duty of care over that of the clinician—such an ‘extreme case’ has been well described by Molnár-Gábor who notes that then, ‘medical liability would in principle be ruled out and the manufacturer’s liability alone would be decisive’.^53^

##### b. Breach of Duty

Although it seems unlikely, if the duty of care question is resolved in favour of the plaintiff, they would then need to prove on the balance of probabilities that the software developer had breached this duty. Mason J in *Wyong Shire Council v Shirt*^54^ set out matters to be considered in determining this: ‘[t]he perception of the reasonable man's response [to the risk of harm to the plaintiff] calls for a consideration of the magnitude of the risk and the degree of the probability of its occurrence, along with the expense, difficulty and inconvenience of taking alleviating action and any other conflicting responsibilities which the defendant may have’.^55^ Applying this to the scenario, a finder of fact might determine that the risk of harm to the patient from the faulty CDSS was high although the likelihood of it occurring was low due to the anticipated intervention of the clinician. Although further testing of the software algorithm before deployment to minimise the risk would not be without cost, a court would likely consider it to be a reasonable step. Maintaining a clear record of the software development, testing and maintenance process would be a useful pre-emptive defensive measure by medical software developers.^56^ So would communicating to users the source and currency of the guidance underlying the algorithms, and the appropriate degree of reliance on them. Overall, if a duty of care was established, it is likely that a court would find that in the given scenario the software developer had breached their duty of care through insufficient testing of the algorithm and through failing to warn the clinician not to rely wholly on the CDSS recommendations.

##### c. Defence of Intermediate Examination

A software developer subject to a negligence claim by a patient’s estate may raise the defence of ‘intermediate examination’, noted by Lord Atkin in *Donoghue v Stevenson*. In that case, whereby a manufacturer was held liable for a decomposed snail in a bottle of ginger beer that harmed the ultimate consumer of the product, the famous bottle was opaque, meaning that the snail’s remains ‘were not, and could not be, detected until the greater part of the contents of the bottle had been consumed’.^57^ Had it been possible for someone along the supply chain to have inspected the product, this may have vitiated the duty of care otherwise existing between the manufacturer and ultimate consumer. Still, case law indicates that the manufacturer (or other third party) only avoids liability in this way if they had a reasonable expectation that the intermediate inspection would be ‘an adequate safeguard to persons who might otherwise suffer harm’.^58^ The defendant software developer would need to demonstrate on the balance of probabilities that they had a reasonable expectation that the clinician using the CDSS would intercept any errors that would otherwise place the patient at risk. It is not enough that there merely be an *opportunity* for intermediate examination.^59^ This defence seems unlikely to succeed; the defendant software developer would be in a much stronger position in this regard had they included suitable warning or instructions to the clinicians using the product.

#### *2.* Patient’s Claim for Breach of ACL Section 138: Goods with Safety Defects

In addition to a negligence claim, a further cause of action against the software developer is available to the patient’s estate under section 138 of the ACL, whereby:

1. A manufacturer of goods is liable to compensate an individual if:
  - The manufacturer supplies the goods in trade or commerce; and
  - The goods have a safety defect; and
  - The individual suffers injuries because of the safety defect.

The fact that the software developer, as ‘manufacturer’, has not supplied the software to the patient directly in the scenario is unproblematic here—as long as it was supplied to a person in the contractual chain.^60^ This is significant because it provides a clear pathway for legal action by the patient’s estate against the software developer; a pathway which may otherwise be unavailable or at least disputable under the law of negligence.

In considering potential action under section 138, two elements are important. First, this provision relates only to goods and not services. It is necessary to distinguish which parts of the supply by the software developer fall into each category. In the 2014 matter of *Goldiwood Pty Ltd v ADL*,^61^ Adjudicator Gordon categorised the supply of software and regular development and enhancements of the software through new releases as ‘goods’, whilst bug fixes and workarounds specific to an individual user under a contractual obligation to provide support would be ‘services’. Secondly, it must be shown that the patient’s death was ‘because of’ the software error, taking a common-sense approach to interpreting those words.^62^ If the patient’s estate could show that the defect in the software was a ‘necessary condition’ of the patient’s death, this would suffice.^63^

Would the type of software problem described in the scenario qualify as a ‘safety defect’ under section 138(1)(b) of the ACL? The term is defined in section 9 of the ACL: ‘goods have a safety defect if their safety is not such as persons generally are entitled to expect’, taking into account ‘all relevant circumstances’ including marketing, packaging, instructions and warnings. Goods need not be absolutely risk-free; rather, their safety is assessed by taking into account the ‘objective knowledge and expectations of the community’.^64^ Would the software be considered ‘as safe as persons generally are entitled to expect’ (not examining whether the software developer’s conduct was ‘reasonable’)?^65^ There is at least a possibility that our hypothetical software developer’s product would fail this test, particularly in the absence of a warning label or statement about the extent to which its advice should be relied upon.

The statutory defences provided in section 142 of the ACL would not be relevant to the defence case, other than perhaps the so-called ‘state-of-the-art’ defence, whereby the defect could not be discovered given the state of scientific or technical knowledge at the time of supply (section 142(c)).

As to remedies, if a claim under section 138 of the ACL succeeds, the patient’s estate can be awarded damages, assessed as under a negligence action.^66^

#### *3.* Rights as a Third Party under the Contract between Clinician and Software Developer

Since the contract for the sale and purchase of the CDSS will involve the software developer and clinician or health service, the privity doctrine prevents a patient from pursuing the software developer for breach of contract. It is unlikely that any such contract would contain a reference to patients as third parties gaining contractual rights or intended to directly benefit from the contract. There is some emerging case law in England whereby a contractual party can sue to recover losses suffered by a third party if the third party has no cause of action of its own against the defendant, but *only* if when the contract was made, ‘there was a common intention and/or a known object to benefit the [third party] or a class of persons to which [the third party] belonged’.^67^ Were this precedent to apply in Australia,^68^ it is still unclear whether a relevant ‘common intention’ between the software developer and clinician as the contracting parties could be made out. In any case, the existence of a cause of action by the patient’s estate against the software developer under the ACL appears to rule out this option.

### C. *Summary: Potential Claims against Clinician and Software Developer by Patient’s Estate*

In summary, this analysis has shown that a patient would likely have a successful claim in negligence against the clinician. Asserting the clinician’s reliance on faulty software is unlikely to be an effective defence. The patient could also bring a successful action under section 138 of the ACL relating to defective goods, although this depends on their overcoming the ‘causation’ challenge.

It is less likely that a patient would be able to establish sufficient proximity between themselves and the software developer to succeed in a negligence action against the software developer directly. There is probably no basis for the patient to join an action for breach of contract between the software developer and clinician. This leads to the next piece of the liability puzzle: considering claims by the clinician against the software developer.

## V. CLAIMS BY THE CLINICIAN

In the scenario being considered, a clinician uses a CDSS which contains a faulty algorithm generating inappropriate recommendations. As a result of the clinician having followed the system recommendations in prescribing a certain medication, their patient dies. As well as the patient’s negligence claim against them, the clinician may be subject to disciplinary proceedings for breaching their professional obligations. It was noted earlier that the clinician may suffer psychological harm and consequential economic loss, for instance through adverse publicity or restrictions on their clinical practice, although they are not harmed directly by the faulty software. The following sections will outline potential avenues for legal redress by the clinician. It is important to note that while this article considers claims in negligence and breach of the consumer guarantees in the ACL, both may be excluded by the contract.^69^

### A. *Clinician Claim in Negligence Against Software Developer*

#### *1.* Duty of Care

Although Australian courts have often considered the scope of product liability, there appear to be no reported cases in Australia specifically considering a duty of care owed by software developers to software users.^70^ This lack of precedent makes it uncertain whether a duty between software developer and clinician would derive from the existing category of manufacturer–consumer duty described in *Donoghue v Stevenson*,^71^ or whether a ‘novel duty’ would need to be established. In considering the former (a view adopted by at least one author^72^), it would be necessary to show that the software developer knew that a failure to take reasonable care would cause injury to the consumer’s person or property.^73^ While this may not be easy to establish, it is possible if the harm to the consumer (clinician) crosses the legal threshold of a ‘recognised psychiatric illness’.^74^

As an alternative to relying on the manufacturer-consumer duty, a clinician taking action in negligence against a software developer may seek to establish a ‘novel duty’, referring to the ‘salient features’ identified in *Caltex Refineries* as outlined above. Examining the relevant salient features, a court would weigh the foreseeability that a failure to take reasonable care in the design and testing of a CDSS could cause harm to the clinician. It would consider the nature of the harm allegedly suffered by the clinician, namely psychological harm (if it meets the definition of a ‘recognised psychiatric illness’), and consequential economic loss, as well as the fact that the clinician was able to exercise a significant degree of control to avoid the harm. In fact, the classification of CDSS as ‘decision support’ tools demands that clinicians exercise independent decisional control in using the software, and they assume responsibility for their actions in delivering patient care. Likewise, the degree of reliance by the clinician upon the software developer may be substantial but is far from absolute; they will also rely upon their training, experience and various other sources of information including the patient’s medical record. The clinician’s vulnerability to harm from the software developer’s conduct is constrained by the clear expectation that the clinician will take steps to protect themselves. In considering the final ‘salient feature’ outlined by Allsop J, the court would also weigh up the desirability of establishing a duty of care in terms of the need for ‘conformance and coherence in the structure and fabric of the common law’. As indicated earlier, the wide-ranging duty of care that clinicians owe their patients may be impinged upon by creating a new duty of care owed by developers of medical software to clinicians. While, as always, the facts of an individual case will be key, it seems unlikely that a novel duty of care would be established based on the given scenario.

#### *2.* Breach of Duty

If a duty of care *is* found to exist between the software developer and the clinician, there would be little difficulty in showing that the software developer had breached this duty (with reference to *Wyong*). The magnitude of the risk of harm to the clinician is high; it would have been straightforward for the algorithms to be tested before deployment in clinical care and for information about the software and the appropriate degree of reliance to be provided to the user; and the ‘reasonable software company’ would have taken such a step.

#### *3.* Causation

A defendant software developer may assert that the clinician’s action in wrongly prescribing two beta-blockers constituted a *novus actus interveniens* breaking the causative chain. Whilst on one view, this accords well with the position that liability for the patient’s harm properly rests with the clinician, on the other, it can hardly be said that the clinician’s act is entirely ‘voluntary’ or ‘causally independent’.^75^ As noted by Mason CJ in *March v E & MH Stramare Pty Ltd*, ‘it makes no sense to regard the negligence of the plaintiff or a third party as a … *novus actus interveniens* when the defendant’s wrongful conduct has generated the very risk of injury resulting from the negligence of the plaintiff or a third party and that injury occurs in the ordinary course of things’.^76^ Yet reframed in light of the Victorian statute, with a focus on policy, while it is likely that the software developer’s negligence was a ‘necessary condition of the occurrence of the harm’, there is a real question as to whether it is ‘appropriate for the scope of the negligent person’s liability to extend to the harm so caused’.^77^ These policy considerations will be addressed further in the Discussion.

#### *4.* Contributory Negligence

Under the apportionment legislation,^78^ if the software developer is found to have acted negligently a court may nonetheless find that the plaintiff clinician’s damage was caused partly by their failure to take reasonable care for their own safety. Liability would be apportioned between the parties, with a potential reduction in damages of up to 100% (hence defeating the claim) if the court considers it ‘just and equitable to do so’.^79^ What the clinician might have done to avoid the harm, particularly in the cross-checks that might have been undertaken to identify and overcome the software’s erroneous prescribing alert, would be the focus of argument on this issue, as would the extent to which the risk was obvious and the amount of instruction the clinician had received in the use of the software.^80^ On the given facts, a finding of contributory negligence of the clinician seems likely.

### B. *Clinician Claims against Software Developer under Consumer Protection Law*

As outlined above (Section IV(B)), separate to a potential action in negligence the ACL offers a vehicle for holding software developers to account for faulty software. Clinics and individual clinicians purchasing a software licence are protected under the ACL, with such licences likely to be treated as ‘goods’ under the law.^81^ This applies if the software is supplied by a business (a ‘corporation’^82^); whilst supply by individuals can also be captured either under the ‘telegraphic or telephonic services’ provisions of the *Competition and Consumer Act 2010* (Cth) (section 6(3)) or state- and Territory-based applications of the ACL.^83^ Businesses that purchase goods costing up to $100,000 are treated as ‘consumers’ under the ACL,^84^ and certain statutory ‘consumer guarantees’ apply to these goods. Two important guarantees are that goods must be ‘of acceptable quality’ (ACL section 54); and fit for purpose (ACL section 55), which are now considered.

#### *1.* Acceptable Quality (Section 54(1))

‘Acceptable quality’ is defined by the expectations of a hypothetical reasonable consumer.^85^ The most relevant elements of this definition are: ‘fit for all the purposes for which goods of that kind are commonly supplied’, ‘free from defects’, and ‘safe’.^86^ Importantly, this is not a guarantee that a product as supplied will be perfect—as Paterson notes, ‘Rather, it is a guarantee that the goods are of a quality that a reasonable consumer would consider acceptable, taking into account the circumstances of the particular transaction’.^87^ Nor must goods be absolutely free from defects or entirely safe. Relevant defects might include those that are inherent as well as defects of design and inadequate or faulty instructions.^88^

In the given scenario it is possible that the product would fail to meet the ACL guarantee of acceptable quality, although as Burrows has noted, our hypothetical reasonable clinician–consumer is likely to be at least conceptually aware that such software can contain bugs.^89^ Moreover, there are specified matters to be taken into account in assessing ‘acceptable quality’ under the ACL; these are the nature and price of the goods, statements on their packaging and representations made about them by the supplier/manufacturer, and ‘any other relevant circumstances’ (section 54(3)(e)). These considerations will be especially relevant to an assessment of the quality of CDSSs, since it will be inherent in their description that they are designed to provide ‘decision support’ rather than to be relied upon unquestioningly. Certainly, in *Goldiwood v ADL* it was held that software with three specified defects did not fail the section 54 ‘acceptable quality’ provision of the ACL, with Adjudicator Gordon noting ‘I accept that the software was capable of providing *substantially* the functionality for which it was supplied … *It is inevitable that software will have some problems*’.^90^ Hence, it may be difficult to succeed in a claim under the ACL if the algorithmic defects in a CDSS product are relatively minor or narrowly confined within the context of a broad range of effective functionality. Notably, however, *Goldiwood* was not about medical software; a product type where the standard for ‘acceptable quality’ may be higher. What amounts to tolerable error rates within CDSSs remains uncertain, though the degree of risk attached to particular types of CDSS functions may be relevant. For instance, recommendations for prescribing or pathology testing related to acute or potentially life-threatening disease or high-risk medications may be judged according to a higher standard than reminders for recall, general information provision or preventive health care issued by such a system.

#### *2.* Fitness for Purpose (Section 55(1))

Section 55(1) of the ACL provides a guarantee that goods supplied to a consumer (except at auction) ‘are reasonably fit for any disclosed purpose, and for any purpose for which the supplier represents that they are reasonably fit’. ‘Disclosed purpose’ is defined in section 55(2). Again, this is an objective test. Absolute fitness for purpose is not required. This provision sets a higher quality standard than the guarantee of acceptable quality in section 54.^91^ In determining whether a medical software product such as a CDSS satisfies the guarantee of fitness for purpose, it will be important to consider the facts as to any specific communication between the software supplier and the purchasing doctor or clinic. For instance, a doctor’s indication (express or implied) that they wanted software able to identify with absolute accuracy all patients meeting certain risk criteria would be relevant to the determination of whether the guarantee of fitness for purpose has been met in a specific product. In *Goldiwood*, certain defects in the software were not found to breach section 54 (acceptable quality) but were found to breach section 55 (fitness for purpose) because ‘these were things that [the purchaser] explained they needed from the system, yet they were not part of the functionality of the software upon delivery and installation’.^92^ This highlights the critical importance of both parties clarifying and communicating the degree of appropriate reliance on CDSSs at the outset.

#### *3.* Remedies

Upon a successful claim under the ACL, a range of statutory remedies is available (independent of contractual remedies). Remedies for failing to meet the consumer guarantees include repair, replacement or refund, and compensation for consequential loss, under section 259 of the ACL.^93^ For the purposes of this analysis, we are most interested in the latter remedy, which is available when any loss or damage is ‘reasonably foreseeable’ to result from a failure to comply with guarantees.^94^ A causal connection between the failure and the loss is required,^95^ akin to the test of remoteness in negligence. While section 259(4) is broad in scope—referring to ‘any loss or damage suffered by the consumer…if it was reasonably foreseeable that the consumer would suffer such loss or damage as a result of such a failure’ [to comply with the guarantee]—it is not yet settled law whether damages for mental harm arising from the failure to comply with consumer guarantees is available.^96^ However, the software developer’s liability under the consumer guarantees can be reduced by contractual terms that limit remedies to replacement or repair of goods (unless reliance on such exclusion was considered not ‘fair or reasonable’).^97^

### C. *Clinician Claim for Breach of Contract*

The clinician or their employing health service which purchased the software may have a claim for breach of contract, depending on the specific terms of the contract. This can provide a remedy for pure economic loss as well as personal injury.^98^ An action for breach of contract may be in addition to a claim for statutory remedies.^99^ This is not considered further here, as the particular terms of any contract will be determinative.

## VI. REGULATORY RESPONSES

In addition to personal actions against the clinician and the software developer in this arena, there is a role for regulators in responding to harms caused by faulty CDSS. As discussed below, neither the ACCC nor the TGA has taken a particularly interventionist approach regarding CDSS to date.

### A. *ACCC*

The ACCC and relevant state or territory regulators can take a representative action under section 149 of the ACL for defective goods supplied by the manufacturer for which a cause of action has arisen under section 138. In practice, the likelihood of this seems remote, at least at present, given the ACCC’s stated focus on litigation ‘where the conduct is by a large or national trader or results, or has the potential to result, in competitive harm or substantial consumer or small business detriment’.^100^

### B. *TGA*

#### *1.* Overview of Regulation of CDSS

Problems with medical software are more likely to be highlighted by the TGA than the ACCC. Even there, however, the clinician’s role in exercising independent decision-making in relation to the software-generated recommendations helps to shield medical software developers from regulatory attention. Some CDSSs fall outside TGA regulation altogether; for instance, those systems intended only to display clinical guidelines or recommendations.^101^ Other types of software that may have functionality similar to decision support, such as risk calculators and alerts, are also expressly excluded from TGA regulation.^102^

The TGA issued new advice in 2021 that ‘exempts’ certain CDSS from registration if the software meets the following three criteria:

- intended by its manufacturer to be for the sole purpose of providing or supporting a recommendation to a health professional about preventing, diagnosing, curing, or alleviating a disease, ailment, defect or injury in persons; and
- not intended by its manufacturer to directly process or analyse a medical image or signal from another medical device; and
- not intended by its manufacturer to replace the clinical judgement of a health professional in relation to making a clinical diagnosis or decision about the treatment of patients.^103^

‘Exemption’ essentially places these products under a light-touch regulatory regime whereby the TGA still retains some oversight of these products in relation to adverse events, advertising, and notification.^104^ Importantly, the TGA must be notified of the software,^105^ and the software must still comply with relevant ‘Essential Principles’ set out at Schedule 1 of the *Therapeutic Goods (Medical Devices) Regulations 2002*.^106^ These include the following.

- Devices are not to compromise patient health and safety (Principle 1).
- Design and construction must conform with safety principles having regard to the state of the art (Principle 2).
- Design and production must ensure safety over the product life (Principle 4).
- The product’s benefits must outweigh any undesirable effects (Principle 6).
- Design and production ensure that the ‘safety, performance, reliability, accuracy, precision, useability, security and repeatability of the device are appropriate’ for its intended purpose, and any risks associated with fault conditions are ‘appropriately reduced’ (Principle 12.1(1)).
- Software integrated with computing platforms ‘must be designed and developed taking into account the capability, resources and configuration of the platforms and external factors’ (Principle 12.1(3))
- Detailed instructions must be provided with the software (Principle 12.1(4)).
- There must be protection against cybersecurity risks (Principle 12.1(5)).
- Certain information must be provided with the software (listed in Principles 13.3 and 13.4) that identifies the software (including version number and build number) and manufacturer (Principles 13.1(1) and 13B(1)).
- ‘Every medical device requires clinical evidence, appropriate for the use and classification of the device, demonstrating that the device complies with the applicable provisions of the essential principles’ (Principle 14).

In addition, CDSSs which are treated as ‘exempt’ must state the basis of their recommendations, including sources, sufficient to enable independent review.^107^

Although the *Therapeutic Goods (Medical Devices) Regulations 2002* also states that CDSS manufacturers must apply the appropriate conformity assessment procedures (schedule 4, part 2, Item 2.15 ‘Conditions’ (b))—in fact no such procedures are stipulated for CDSSs.^108^ The manufacturer must notify the Secretary about adverse events (which includes non-compliance with the Essential Principles^109^) within certain timeframes. The TGA can issue a recall or a hazard alert in relation to these systems.^110^

#### *2.* Consequences of TGA Regulatory Regime for ‘Exempt’ CDSSs

The regulatory regime emphasises the manufacturer’s self-assessment against the Essential Principles. There are significant criminal and civil penalties for supply of a medical device that fails to comply with these Principles, under sections 41MA and 41MAA of the *Therapeutic Goods Act 1989* (Cth) including up to 5 years imprisonment where harm or injury (connected with non-compliance with the Essential Principles) has resulted or is likely to result from use of the device. Conversely, if a software developer is subject to the types of legal action by patients or clinicians outlined earlier, being able to demonstrate that they complied with the TGA’s Essential Principles ought to have a legally protective effect; it could help to prove that reasonable care had been taken regarding device safety and reliability.

While health care professionals can report adverse events relating to CDSS to the TGA, the effects of such reporting are uncertain. The TGA states that following a report of an adverse event, ‘isolated incidents or incidents not likely to lead to injury or a detrimental effect to patients or operators are not routinely investigated’.^111^

In summary, the software developer in the scenario in section III is subject to a comparatively light-touch regime under the *Therapeutic Goods Act 1989* (Cth), being exempt from the requirement to register their product on the Australian Register of Therapeutic Goods. The likely classification of many CDSS as ‘exempt’ under the regulatory scheme emphasises clinicians’ need to exercise appropriate judgement in utilising the recommendations that emerge from such systems. The Essential Principles do provide a valuable self-assessment framework, compliance with which may assist the software developer to avoid causing harm in the first place and to defend themselves against legal claims in the second. Failure to comply with the Essential Principles can also result in significant criminal and civil penalties under the legislation.

## VII. DISCUSSION

This article has analysed in detail what recourse clinicians and patients might have, and what actions regulators might take, against a software developer supplying a CDSS flawed in a particular way that generates incorrect clinical guidance leading to harm, in the Australian context. A key finding of this analysis concerns the challenge facing the clinician, whose duty of care to the patient essentially increases their own legal risk if they place undue reliance on CDSSs. Due to this overriding and wide-ranging duty of care, it is unlikely that the clinician in the scenario would successfully defend the patient’s claim either in negligence, contract or under the ‘due care and skill’ consumer guarantee in the ACL. This is irrespective of the fact that it was the software that first introduced the error. Heightened awareness of the potential flaws of medical software and a sense of diligence in cross-checking information sources will thus be needed. It will also be important that clinicians satisfy themselves as to a particular system’s credentials, including the source and currency of substantive guidelines, and quality assurance processes. Completed TGA ‘Essential Principles’ documentation would provide a valuable resource in this task.

Difficulties with making out a duty of care and causation will hinder potential claims by the patient against the software developer directly, irrespective of the cause of action. Their clearest route likely lies in the defective product provision of the ACL (section 138), but whilst this overcomes the lack of direct relationship between the patient and the software company, establishing causation still presents an obstacle to the patient’s claim.

The clinician, meanwhile, is most likely to bring a successful claim against the software developer under the terms of the contract and the consumer guarantees in the ACL. The size of any award may be minimal, however. Evidence as to the harm suffered by the clinician will be relevant here.

Software developers supplying CDSS containing algorithmic errors that generate incorrect recommendations leading to harm are most likely to face claims from clinicians under the terms of the contract and provisions of the ACL. What should they do to protect their interests? First, as suggested by the scenario itself, the use of a range of quality assurance measures including effective testing, monitoring and standard operating procedures, all with up-to-date written documentation, should be prioritised within organisations developing medical software. This is particularly the case where software generates recommendations designed to impact on patient care. Secondly, communication with software users, both before and after purchase, should specify the source and currency of the recommendations and the importance of installing available updates. It should also make it clear that despite the quality assurance measures that have been implemented, clinicians should not rely solely on the CDSS recommendations in providing health care to patients. Thirdly, software developers whose CDSS products are ‘exempt’ under the TGA’s regulatory scheme must still comply with the TGA’s Essential Principles and document this. Finally, they should take account of the probability that over time, as technology—particularly artificial intelligence—advances and algorithmic impenetrability increases, the likelihood of errors increases and their risk of being subject to a successful claim in negligence will grow. The greater degree of reliance that is intended to be placed in the software, the greater the chance that a clear duty of care will be found to exist.

This analysis calls into question TGA’s recent clarification that CDSSs meeting certain criteria are ‘exempt’, including from the requirement to be listed on the Australian Register of Therapeutic Goods. While the exemption accords with the position reached in this analysis—that is, in the context of CDSS use, the duty to avoid harm to patients falls squarely on clinicians—a real question of fairness is emerging. This software is, by its nature, built to be relied upon in clinical decision-making, and has many clear benefits in terms of reducing overall error rates, improving the quality and consistency of health care provision and alignment of care with clinical practice guidelines. Given that the TGA has only recently announced its position regarding CDSSs, it remains to be seen whether the new requirement to comply with and document software systems against the ‘Essential Principles’ will be sufficient to focus attention on, and to drive up, these products’ quality, safety, and transparency. Will it be sufficient response to the Royal Australian College of General Practitioners’ position that ‘[t]he current unregulated environment creates significant risks for practitioners due to the varying quality and currency of information, as well as the lack of consistency across different software’?^112^ Ideally, the TGA will monitor closely the new regulatory approach, especially software developers’ acquittal of the ‘Essential Principles’ documentation, which I propose should be mandatorily published, or at least supplied to prospective purchasers.

## VIII. CONCLUSION

Despite all the evidence of the clinical benefits of CDSSs, their use can also result in real harm to health care recipients. The extent of CDSSs’ promulgation across whole health care ecosystems raises the potential of this harm to occur on a widespread scale. Despite this, both the current regulatory approach and the contour of legal risk still centre upon the clinician’s duty to exercise decisional autonomy and to intercept flawed recommendations generated by algorithmic errors within CDSSs. The growing penetration of CDSS into all manner of clinical activities and the degree of reliance that the products themselves encourage, demands a reframing in both areas. Patients who suffer harm in these circumstances have clear recourse against the clinician, less so against the software developer. Yet, the increasingly clear policy tensions in this area need greater attention and resolution. Health services purchasing CDSSs should be cautious about product claims and clear with the product users about the possibility of flaws and any risk mitigation steps that should be followed. How should clinicians proceed, when they are both encouraged to rely upon the machine and warned against trusting it? Both trust and reliance may be misplaced until a fairer allocation of legal liability and a higher degree of regulatory oversight are achieved.

## Footnotes

1 Megan Prictor and others, ‘Clinical Decision Support Systems and Medico-Legal Liability in Recall and Treatment: A Fresh Examination’ (2020) 28 Journal of Law and Medicine 132, 132.

2 ibid 139.

3
*Rogers v Whitaker* [1992] HCA 58, (1992) 175 CLR 479.

4 Reed T Sutton and others, ‘An Overview of Clinical Decision Support Systems: Benefits, Risks, and Strategies for Success’ (2020) 3 npj Digital Medicine 1, 1.

5 Prictor and others (n 1).

6 I use the term ‘software developer’ in this analysis to mean the organisation producing the software; whilst noting that individual staff within this organisation may be jointly and severally liable with their employer. In determining legal liability for harm, it will be important to examine whether a staff member acted in their own right instead of in accordance with their employer’s instructions.

7 Helen Smith and Kit Fotheringham, ‘Artificial Intelligence in Clinical Decision-Making: Rethinking Liability’ (2020) 20 Medical Law International 131.

8 In relation to medical negligence law, I refer to the Victorian statute (*Wrongs Act 1958* (Vic)).

9 Mirela Prgomet and others, ‘Impact of Commercial Computerized Provider Order Entry (CPOE) and Clinical Decision Support Systems (CDSSs) on Medication Errors, Length of Stay, and Mortality in Intensive Care Units: A Systematic Review and Meta-Analysis’ (2017) 24 Journal of the American Medical Informatics Association 413.

10 Amit X Garg and others, ‘Effects of Computerized Clinical Decision Support Systems on Practitioner Performance and Patient Outcomes: A Systematic Review’ (2005) 293 Journal of the American Medical Association 1223, 1226.

11 Tiffani J Bright and others, ‘Effect of Clinical Decision-Support Systems: A Systematic Review’ (2012) 157 Annals of Internal Medicine 29, 31.

12 ibid.

13 Kaveh G Shojania and others, ‘The Effects of On‐screen, Point of Care Computer Reminders on Processes and Outcomes of Care’ [2009] Cochrane Database of Systematic Reviews. <https://doi.org/10.1002/14651858.CD001096.pub2>.

14 Adam Wright and others, ‘Analysis of Clinical Decision Support System Malfunctions: A Case Series and Survey’ (2016) 23 Journal of the American Medical Informatics Association 1068.

15 Joan S Ash and others, ‘Some Unintended Consequences of Clinical Decision Support Systems’ [2007] AMIA Annual Symposium Proceedings 26.

16 Heleen van der Sijs and others, ‘Overriding of Drug Safety Alerts in Computerized Physician Order Entry’ (2006) 13 Journal of the American Medical Informatics Association 138, 138.

17 Ash and others (n 15) 29.

18 ibid 28.

19 Ash and others (n 15); Kirsten Colpaert and others, ‘Impact of Computerized Physician Order Entry on Medication Prescription Errors in the Intensive Care Unit: A Controlled Cross-Sectional Trial’ (2006) 10 Critical Care R21.

20 Ash and others (n 15) 27.

21 Notably, errors of clinical data that a CDSS is also utilising may result in faulty recommendations (for instance, if a patient’s birth date, gender or test results have been entered incorrectly). Such errors, whilst important, are not the focus of this analysis.

22 JaeHo Lee and others, ‘Impact of a Clinical Decision Support System for High-Alert Medications on the Prevention of Prescription Errors’ (2014) 83 International Journal of Medical Informatics 929.

23 ibid 937.

24 Erin G Stone, ‘Unintended Adverse Consequences of a Clinical Decision Support System: Two Cases’ (2018) 25 Journal of the American Medical Informatics Association 564, 566.

25 Wright and others (n 14) 1068.

26 Richard H Epstein and Franklin Dexter, ‘Unintended Consequences of Clinical Decision Support’ (2019) 128 Anesthesia & Analgesia e124.

27 Stone (n 24).

28 If, for instance, reference to specific clinical guidelines and their date was given, it might be easier for a clinician to make an independent judgement about reliance on the software alerts.

29
*Wrongs Act 1958* (Vic) ss 16–17.

30
*Rogers v Whitaker* (n 3); *Wrongs Act 1958* (Vic) s 48.

31 Smith and Fotheringham (n 7) 134–35.

32
*Wrongs Act 1958* (Vic) s 49(1)(a).

33 ibid s 48(2)(a).

34 ibid s 48(2)(c).

35 ibid s 59(1).

36 This approach is supported by Fruzsina Molnár-Gábor, ‘Artificial Intelligence in Healthcare: Doctors, Patients and Liabilities’ in Thomas Wischmeyer and Timo Rademacher (eds), *Regulating Artificial Intelligence* (Springer International Publishing 2020) 350 <https://doi.org/10.1007/978-3-030-32361-5_15> accessed 3 September 2021.

37
*Breen v Williams* [1996] HCA 57, (1996) 186 CLR 71, [3].

38 Ben White, Fiona McDonald and Lindy Willmott, *Health Law in Australia* (Thomson Reuters 2018) 380 <http://ebookcentral.proquest.com/lib/unimelb/detail.action?docID=5452478> accessed 3 September 2021.

39 The Australian Consumer Law is enacted as Schedule 2 to the *Competition and Consumer Act 2010* (Cth) (‘ACL’).

40 White, McDonald and Willmott (n 38) 383.

41
*Moore v Scenic Tours Pty Limited (No 2)* [2017] NSWSC 733, [425].

42 ACL 267(4).

43 Bill Madden, ‘Medical Claims and the ACL: A Few Thoughts on Exclusion Clauses and Waivers’ (5 July 2018) <https://www.mondaq.com/australia/consumer-law/716232/medical-claims-and-the-acl-a-few-thoughts-on-exclusion-clauses-and-waivers> accessed 1 September 2021.

44
*Donoghue v Stevenson* [1932] AC 562.

45
*Australian Knitting Mills Ltd v Grant* [1933] HCA 35, (1933) 50 CLR 387.

46 Malcolm Burrows, ‘Are Software Developers Liable for Defects in Their Software?’ (*Dundas Lawyers*, 20 December 2016) <https://www.dundaslawyers.com.au/are-software-developers-liable-for-defects-in-their-software/> accessed 2 August 2021.

47
*Caltex Refineries (Qld) Pty Ltd v Stavar* [2009] NSWCA 258.

48 ibid.

49 James Plunkett, ‘Chapter 3: Methods for Determining the Existence of a Duty of Care’ in James Plunkett (ed), *The Duty of Care in Negligence* (Hart Publishing 2018) <https://www.bloomsburycollections.com/book/the-duty-of-care-in-negligence> accessed 1 February 2021.

50
*Wyong Shire Council v Shirt* [1980] HCA 12, (1980) 146 CLR 40, [13].

51 Smith and Fotheringham (n 7) 137.

52
*Wyong Shire Council v Shirt* (n 50); *Home Office v Dorset Yacht Co Ltd* [1970] UKHL 2.

53 Molnár-Gábor (n 36) 348.

54
*Wyong Shire Council v Shirt* (n 50).

55 ibid [14].

56 Anne Fuller and Peter Croll, ‘Why Don’t We Teach Software Engineers about the Law?’ (2001) 12 Journal of Law, Information and Science 139, 147.

57
*Donoghue v Stevenson* (n 44).

58
*Jull v Wilson and Horton* [1968] NZLR 88 (Richmond J).

59
*Griffiths v Arch Engineering Co (Newport) Ltd* [1968] 3 All ER 217; *Herschthal v Stewart & Ardern Ltd* [1939] 4 All ER 123.

60
*Spittles v Michael’s Appliance Services Pty Ltd* [2008] NSWCA 76, [14]; *Cheong v Wong* (2001) 34 MVR 359.

61
*Goldiwood Pty Ltd t/as Margaret Franklin & Associates v ADL (Aust) Pty Ltd t/as Adviser Logic* [2014] QCAT 238, [35]–[40].

62
*Carey-Hazell v Getz Bros & Co (Aust) Pty Ltd* [2004] FCA 8563, [195].

63
*Merck Sharp & Dohme (Australia) Pty Ltd v Peterson* [2011] FCAFC 128, [104].

64 ‘Explanatory Memorandum to the Trade Practices Amendment (Australian Consumer Law) Bill (No. 2) (Cth)’ (2010) 6.

65 Phillip Clarke and Sharon Erbacher, *Australian Consumer Law: Commentary & Materials* (Thomson Reuters 2018) 964 <http://ebookcentral.proquest.com/lib/unimelb/detail.action?docID=5452523> accessed 11 August 2021.

66 Jeannie Paterson, *Corones’ Australian Consumer Law* (Thomson Reuters 2019) 516 <http://ebookcentral.proquest.com/lib/unimelb/detail.action?docID=5837843> accessed 10 August 2021; *Wrongs Act 1958* (Vic) s 16.

67
*BV Nederlandse Industrie Van Eiprodukten v Rembrandt Enterprises, Inc* [2019] EWCA Civ 596; John Gooley, Peter Radan and Ilija Vickovich, *Principles of Australian Contract Law* (LexisNexis Butterworths 2021) [39.17] <http://ebookcentral.proquest.com/lib/unimelb/detail.action?docID=6458381> accessed 27 August 2021.

68
*Yuan v O’Neill* [2020] SASC 49, [77].

69 Unless such exclusion was an ‘unfair term’ of a small business contract (ACL s 23) or, in relation to the consumer guarantees, the exclusion was not ‘fair or reasonable’ (ACL s 64A(3)(4)).

70 Burrows (n 46).

71
*Donoghue v Stevenson* (n 44).

72 Maurice Jamieson, ‘Liability for Defective Software’ [2001] Journal of the Law Society of Scotland <https://www.lawscot.org.uk/members/journal/issues/vol-46-issue-05/liability-for-defective-software/> accessed 2 February 2021.

73 Stephen Watt, 'Defendant's Duty of Care', Halsbury's Laws of Australia (7 June, 2021) accessed 29 October 2021.

74
*Wrongs Act 1958* (Vic) s 72(1); Michael Rowe, ‘Doctors’ Responses to Medical Errors’ (2004) 52 Critical Reviews in Oncology/Hematology 147; Louise Nash, Christopher Tennant and Merrilyn Walton, ‘The Psychological Impact of Complaints and Negligence Suits on Doctors’ (2004) 12 Australasian Psychiatry 278.

75
*Haber v Walker* [1963] VR 339.

76
*March v E & MH Stramare Pty Ltd* [1991] HCA 12, (1991) 171 CLR 506.

77
*Wrongs Act 1958* (Vic) s 51(1), (4).

78 ibid s 26(1).

79 ibid s 63.

80 Stephen Watt, 'Contributory Negligence', Halsbury's Laws of Australia (7 June, 2021) accessed 28 October 2021.

81
*Australian Competition and Consumer Commission v Valve Corporation (No 3)* [2016] FCA 196, (2016) 337 ALR 647; ACL s 2 (definition of ‘goods’). There remains a possibility that a web-based application may be considered a ‘service’ under the Australian Consumer Law; see Burrows (n 46); Benjamin Hayward, ‘What’s in a Name? Software, Digital Products, and the Sale of Goods’ (2016) 38 Sydney Law Review 441, 446.

82 ACL s 4(1) (definition of ‘corporation’), s 130.

83 Paterson (n 65) 56–57.

84 ACL s 3(1)(a)(ii)); Competition and Consumer Regulations 2010 (Cth) reg 77A.

85 ACL s 54(2).

86 ibid s 54(2)(a), (c), (d).

87 Paterson (n 65) 371.

88
*Contact Energy Ltd v Jones* (2009) 2 NZLR 830, [90].

89 Burrows (n 46).

90
*Goldiwood Pty Ltd t/as Margaret Franklin & Associates v ADL (Aust) Pty Ltd t/as Adviser Logic* (n 57) [69]–[70] (emphasis added).

91 ‘Explanatory Memorandum to the Trade Practices Amendment (Australian Consumer Law) Bill (No. 2) (Cth)’ (n 60) [7.43].

92
*Goldiwood Pty Ltd t/as Margaret Franklin & Associates v ADL (Aust) Pty Ltd t/as Adviser Logic* (n 57) [72].

93 Australian Consumer Law, ‘Consumer Guarantees: A Guide for Businesses and Legal Practitioners’ (March 2016) 19 <https://www.accc.gov.au/system/files/Consumer%20guarantees%20-%20A%20guide%20for%20businesses%20and%20legal%20practitioners.pdf> accessed 11 August 2021.

94 ‘Explanatory Memorandum to the Trade Practices Amendment (Australian Consumer Law) Bill (No. 2) (Cth)’ (n 60) [7.95].

95 ACL s 259(4); *Wardley Australia Ltd v Western Australia* [1992] HCA 55, (1992) 175 CLR 514, [11].

96 Paterson (n 65) 687.

97 ACL s 64A(3); Commonwealth of Australia, ‘Consumer Guarantees: A Guide for Businesses and Legal Practitioners’ (2016) 35 <https://www.accc.gov.au/> accessed 11 August 2021.

98 Stephen Watt, ‘'Sources of Product Liability Laws', Halsbury's Laws of Australia (7 June, 2021) accessed 28 October 2021.

99 Anne McNaughton, 'Consequences of Breach of Contract', Halsbury's Laws of Australia (27 September, 2017) accessed 28 October 2021.

100 Australian Competition and Consumer Commission, ‘Compliance & Enforcement Policy & Priorities’ (*Australian Competition and Consumer Commission*, 13 February 2013) <https://www.accc.gov.au/about-us/australian-competition-consumer-commission/compliance-enforcement-policy-priorities> accessed 15 September 2021.

101 Therapeutic Goods Administration, ‘Clinical Decision Support Software: Scope and Examples (Version 1.1)’ (October 2021) 7.

102 These are specified in the *Therapeutic Goods (Excluded Goods) Determination 2018* under s 7AA of the *Therapeutic Goods Act 1989* (Cth). They include software that are risk calculators (Item 14L) and software that is intended to be used solely to provide alerts to clinicians in relation to patient care and is not intended to replace clinical judgement or directly decide about treatment (Item 14I).

103 Therapeutic Goods (Medical Devices) Regulations 2002 (Cth) sch 4, pt 2, item 2.15 (‘Kinds of Medical Devices’ (a)–(c)).

104 Therapeutic Goods Administration, ‘How the TGA Regulates Software-Based Medical Devices (Draft Version 1.1)’ (February 2021) 6.

105 Therapeutic Goods Administration, ‘Clinical Decision Support Software: Scope and Examples (Version 1.1)’ (n 97) 13.

106 Therapeutic Goods (Medical Devices) Regulations 2002 (Cth) sch 4, pt 2, item 2.15 (‘Conditions’ (a)).

107 Therapeutic Goods Administration, ‘Clinical Decision Support Software: Scope and Examples (Version 1.1)’ (n 97) 10.

108 Digital Devices, TGA, ‘Regulation of Software as a Medical Device: Query’ (30 August 2021).

109
*Therapeutic Goods Act 1989* (Cth) s 41MP(2)(c).

110 Therapeutic Goods Administration, ‘Clinical Decision Support Software: Scope and Examples (Version 1.1)’ (n 97) 5.

111 Therapeutic Goods Administration, ‘Overview of How TGA Manages Medical Device Adverse Event Reports’ (*Therapeutic Goods Administration (TGA)*) <https://www.tga.gov.au/overview-how-tga-manages-medical-device-adverse-event-reports> accessed 30 August 2021.

112 ‘RACGP: Electronic Clinical Decision Support in General Practice’ <https://www.racgp.org.au/advocacy/position-statements/view-all-position-statements/clinical-and-practice-management/electronic-clinical-decision-support> accessed 16 September 2021.

## Notes

The author is an investigator on the Future Health Today project, which has a clinical decision support component. The project has been funded in part by the Paul Ramsay Foundation, which contributes to the author’s institutional salary in relation to this project.

